# Children of men with alcohol dependence: Psychopathology, neurodevelopment and family environment

**DOI:** 10.4103/0019-5545.74313

**Published:** 2010

**Authors:** Vijaya Raman, Suveera Prasad, M. Prakash Appaya

**Affiliations:** Department of Psychiatry, St. John’s Medical College Hospital, Bangalore, India; 1 Sheffield Care Trust, Sheffield, United Kingdom

**Keywords:** Alcohol dependent fathers, children, family environment, neurodevelopment, psychopathology

## Abstract

**Background:**

Children of people with alcohol dependence (COAs) are at high risk for behavioral and cognitive problems.

**Aim:**

Aim of this study was to compare the nature and extent of these problems in children of men with and without alcohol dependence.

**Materials and Methods:**

32 children (17 in study group and 15 controls) were evaluated for psychopathology, neurodevelopment, cognitive functioning and family environment. Tools used were: Socio-demographic data sheet, Malin’s Intelligence Scale for Indian Children (MISIC), Child Behavior Checklist, Trail Making Test, Neurodevelopment Scale and the Family Environment Scale.

**Results:**

Children of men with alcohol dependence had higher externalizing than internalizing scores. Children of alcohol-dependent fathers had higher scores on the neurodevelopment scale and lower scores on the performance scale of the MISIC than the children in control group. These children also made more errors on the Trail Making Test. The family environment of COAs was characterized by lack of independence for its members, greater perceived control and lack of adequate cultural and intellectual activities.

**Conclusion:**

Our findings suggest that children of men with alcohol dependence have difficulties with frontal lobe functions and neurodevelopmental tasks. There are also difficulties in the family, which are related to alcohol consumption by the father.

## INTRODUCTION

Parental mental illness and its impact on the development and behavior of children has been an active field of research over the last two decades.

Children of parents with alcohol dependence syndrome are particularly at high risk for substance use as well as other emotional and behavioral problems such as learning disability, hyperactivity, psychomotor delays, somatic symptoms and emotional problems. There have been attempts to study various aspects of children of people with alcohol dependence from India and some published literature is available that looks at various domains in the same sample.[1–4]

Cantwell’s review of prior research indicated that families of hyperactive children have increased prevalence of alcohol-dependent and sociopathic fathers.[5] Six of the seven studies of parents who were alcohol dependent found some association with child hyperactivity. Other childhood conduct problems have also been found such as lying, stealing and truancy.[6–8]

Adolescents who abused alcohol reflected a 46% of parent alcoholism, that is, in 46% of adolescents who abused alcohol, there was family history of alcoholism in the parent/s.[9,10] There was an increased prevalence of physical aggression and low anxiety and this best distinguished sons of male alcohol dependents from normals. Another study concluded that central nervous system hyperexcitability may be etiologically linked to the excess of externalizing behaviors observed in this population, which is thought to be a predisposition to a higher risk of developing early onset alcoholism.[2]

Lieberman, in a review, states that children of alcoholics are two to ten times more likely to develop alcoholism than children of non alcoholics. Children with relatives who abuse or are dependent on alcohol apparently have a slightly higher risk for drug abuse or dependence than those without relatives who consume alcohol.[11] Evidence from twin and adoption studies has highlighted the significance of genetic influences, and the heritability of alcoholism has been estimated at 40–60%[12] Risk factors that mediate the increased vulnerability and protective factors which moderate the risk included in this review are parental antisocial personality disorder, externalizing behavior, internalizing symptoms, differential response to the effects of alcohol and positive and negative alcohol related expectancies.

Neuropsychological functions in these children have been the focus of attention over the last decade. Pihl and Brice[13] reviewed studies of cognitive functioning in children of alcoholics. They found that these children have lower verbal intelligence, overall poor verbal skills, poor verbal and nonverbal memory and poor abstraction and planning. Corral[14] found that children with multi-generational alcoholism, but not children with a father who was alcohol dependent, showed reduced performance in specific cognitive areas such as attention, visuospatial abilities and frontal lobe functions.

Studies have also reported reduced visual event related potential (ERP) amplitudes in young males at high risk for alcoholism. In one study, however, authors found that ERP deviations are not attributable to stages of visual processing deficits but represent difficulty involving more complex utilization of information.[15]

In a comparison of amplitudes and topography of the P300 generated in response to a visual task, between subjects at high risk (offspring of treatment seeking patients with alcohol dependence) and those at low risk for alcoholism (alcohol naïve individuals with absence of family history of alcohol dependence in any of the first- or second-degree relatives) and its relation to externalizing behaviors, Silva and others found that high risk subjects have lower P300 amplitudes over frontal brain areas. They found that differences are greater in young, tending to converge with increasing age. They also described a strong association between this reduced brain activation and an excess of externalizing behaviors in high risk individuals. The authors concluded that a maturational lag in brain development, causing central nervous system disinhibition and externalizing behaviors may underlie the susceptibility to alcoholism.[2]

The highest risk for developing alcoholism exists for individuals who start using alcohol as adolescents, have a high family loading for alcohol problems and display a cluster of behavior traits described as disinhibited, undercontrolled or impulsive, which are usually evident in childhood and persist into adulthood.[16,17]

On the Child Behavior Checklist (CBCL), Christiansen[18] found that in 103 Danish children of alcohol-dependent fathers, there was a significantly greater incidence of symptoms on 17 of the 118 CBCL items. These authors found that daughters of alcohol-dependent fathers were more impaired than sons of alcohol-dependent fathers on most CBCL measures. In a series of investigations, a positive association has been reported between parental alcoholism and impaired emotional functioning of offspring.[19]

Rodriguez Holguin[20] found, in his study, that when compared to controls, children of alcoholics had smaller middle latency auditory evoked potential which the authors opine points to an anomalous pattern of information transmission from the thalamus to cortex.

Rao[1] found that alcoholism was present in parents of malnourished (49.4%) and injured children (45%).

In another study, Stanley and Vanitha[4] investigated the manifestation of self-esteem and adjustment in a group of 50 adolescent children of alcoholic parents and a matched reference group of adolescent children of non-alcoholic parents. The data revealed lower self-esteem and poor adjustment in all the domains studied in the adolescent children of alcoholic parents than the controls. These deficits, the authors opined, can be attributed to the increased stress and vitiated alcohol complicated domestic environment of the children of alcoholic parents. This study made a strong case for psychosocial intervention for these children.

The review of available literature indicates that children of parents with alcohol dependence are at risk for a wide range of neuropsychological dysfunction and psychopathology.

This study was an attempt to examine the broad areas of dysfunction in children of fathers with alcohol dependence in the Indian context so that early identification and intervention can be planned.

## MATERIALS AND METHODS

This study attempted to look at psychopathology, cognitive functioning and family environment in a group of children of men with alcohol dependence and compared them with a control group of children of men without alcohol dependence.

The study was approved by the Hospital Ethics Review Board. Informed consent was taken in writing from all the parents of the children who participated in this study.

The study was conducted in the Department of Psychiatry attached to a tertiary care general hospital.

### Sample

The sample was obtained through referral by one of the co-authors. The patients – both in-patient and out-patient – diagnosed as Alcohol Dependence Syndrome according to ICD 10 criteria (WHO, 1992)[21] and who had children aged between 5 and 9 years (as the Neurodevelopmental Examination is standardized for this population) – both male and female – were included in the study. A control group from the pediatrics out-patient department and the school setting was taken with no parental psychiatric illness and mother scoring less than 4 on the General Health Questionnaire (GHQ).

The inclusion criteria for children were: father diagnosed as Alcohol Dependence Syndrome; having no other known psychiatric illness; aged between 5 and 9 years; with mother having GHQ scores less than 4 and living with the index parent for at least the preceding year.

The exclusion criteria were: parent having any known organic brain syndromes, mental retardation or any other psychiatric illness, children having any known visual/auditory handicap, children with known chronic medical illness like diabetes, asthma or chronic renal disease.

### Procedure

All the assessments were done by the first and second authors who were blind to the clinical status of the father. Subjects for the study were referred by the third author from the out-patient and in-patient services. First, for both the groups, the mother was screened using the GHQ (Goldberg)[22] for psychopathology. Children whose mothers scored above the cut-off on the GHQ were excluded from the study. The fathers in the study group were then administered the Severity of Alcohol Dependence Questionnaire (SADQ)[23] by the second author. The assessments involving the parents, the CBCL and the Family Environment Scale (FES), were administered by the second author and the assessments involving the child, Malin’s Intelligence Scale for Indian Children (MISIC), Neurodevelopmental Examination, and Trail Making Test (TMT), were administered by the first author.

The GHQ is a symptom and well-being scale that serves as a screening for psychopathology. It is a self-rating scale with the severity of symptoms compared to the habitual state of the person in question on a 4-point rating scale. It has sensitivity and specificity of 80–90% in relation to interview-based diagnosis. The 28-item version was used in this study.

The SADQ is a short, easy to complete, self-administered 20-item questionnaire designed to measure severity of dependence on alcohol. There are five subscales with four items in each: Physical withdrawal, Affective withdrawal, Withdrawal Relief Drinking, Alcohol Consumption and Rapidity of Reinstatement. Each item is scored on a 4-point scale, ranging from “Almost Never” to “Nearly Always”, resulting in a corresponding score of 0–3. Thus, the total maximum score possible is 60 and the minimum is 0. Test–retest reliability of this instrument is 0.85 and factor analysis yields single main factor accounting for 53% of variance.

The mother was interviewed by the co-author using a socio-demographic data sheet (developed for the study), the CBCL (Achenbach and Edelbrock)[24] and the FES (Moos).[25]

The CBCL is a descriptive instrument designed to classify behavioral and emotional disorders of children aged 4 through 16 years comprehensively. Each of the 113 items is scored on a 3-step response scale: 0, not true; 1, sometimes true; and 2, often true. The test can be self-administered or administered by an interviewer. The two broad band groupings of behavioral problems assessed correspond to the internalizing and externalizing behavior. Adequate reliability and validity has been established by the authors.

The FES assesses the social environments of families along 10 salient dimensions. It focuses on measurement and description of the interpersonal relationships among family members, on the directions of personal growth emphasized within the family, and on the basic organizational structure of the family. The FES significantly discriminates among families, is sensitive to parent–child differences in the way in which families are perceived, is related to family size and drinking patterns and discriminates between psychiatrically disturbed and matched “normal” families. This scale has a reliability of 0.67 and the validity of this tool has been established.

The author then assessed the children on the MISIC (Indian adaptation of the Wechsler’s Intelligence Scale for Children, Malin).[26] This scale is used widely in testing the intelligence in children of age between 5 and 16 years. It provides a Full Scale IQ, a Verbal IQ and a Performance IQ. The reliability and validity of this instrument is well established.

The TMT (Luria)[27] was then administered. This is a test of controlled attention and is widely used as a screening device, a test of laterality: a test of perceptuo motor functions and is a sensitive measure of frontal dysfunction. The test consists of a set of numbers (1–16) and alphabets (A–P) randomly spread on a sheet of paper. The child is asked to alternate between alphabetical and numerical sequence (1-A, 2-B, etc.). The total time taken and the number of errors made are recorded. The TMT has been found to have a high reliability (0.81) and adequate validity.

The final test done with the children was the Neurodevelopmental Examination (Lindahl *et al*.).[28] This test is used in children of age between 5 and 9 years. This examination consists of 21 tasks. This ranges from an evaluation of the child’s auditory and visual acuity to threading wooden beads within a time limit. It is a sensitive indicator of neurological compromise and is known to pick up subtle deficits. Each item was scored 0, 1 or 2: 0 score if normal, 1 if uncertain or mildly abnormal and 2 if abnormal. The maximum score is 40 and a score of 8 and above is considered significant and indicates dysfunction. The test–retest reliability coefficient of this instrument is established at 0.78.

### Statistical analysis

All the data obtained were analyzed after loading on to SPSS and using the student’s “*t*” test. A comparison of all the parameters – psychopathology, cognitive functions and family environment – was made between the study group and the control group.

## RESULTS

Forty-three children were screened for the study. During the screening procedure, a number of children (*n*=11) had to be excluded. The reasons were: child having had no formal education (*n*=4), child unable to come for completing the assessment (*n*=2) and mothers scoring high on the GHQ (*n*=5). Out of 32 children included in the study, 17 (8 boys and 9 girls) were in the study group and 15 (6 boys and 9 girls) were in the control group. The sample was restricted by the time frame available for the study. As can be seen in Table 1, there were no significant differences between the two groups with respect to age of the child. Table 2 also reveals no differences between the two groups with respect to sex of the child.

**Table 1:** Socio-demographic profile of the sample (age)

	Mean (years)	SD	df	*“t”*	Significance
Age					
COA (*n*=17)	7.6	2.1	30	1.65	NS
CON (*n*=15)	7.9	2.8			

COA - Children of alcohol-dependent fathers; CON - Children of normals

**Table 2:** Socio-demographic profile of the sample (sex)

	Children of alcoholics (*n*=17)	Children of normals (*n*=15)
Age (mean)	7.6 years	7.9 years
Sex		
Male	8	6
Female	9	9

The scores on the CBCL were analyzed on the basis of the two scales – Internalizing Scale and Externalizing Scale. Table 3 shows the scores obtained by the two groups on the two scales of the CBCL.

**Table 3:** Mean scores obtained by the two groups on the CBCL

CBCL	*n*	Mean	SD	df	*“t”*	Significance
Internalizing scale						
COA	17	5.92	2.78	30	1.17	NS
CON	15	4.54	3.1			
Externalizing scale						
COA	17	8.86	4.38	30	3.57	*P*<0.01
CON	15	5.91	5.01			

COA - Children of alcohol-dependent fathers; CON - Children of normals

As can be seen from the table, the children of alcohol-dependent parents obtained statistically significant high scores on the externalizing scale and a marginal, though not statistically significant, high score on the Internalizing Scale.

Table 4 contains the scores obtained on the Neurodevelopmental Examination. There is a statistically significant difference between the two groups on the Neurodevelopmental Examination, with children of alcohol-dependent parents scoring higher, indicating more difficulty with neurodevelopmental tasks.

**Table 4:** Scores obtained by the two groups on the neuropsychological tests – Neurodevelopmental Examination, Malin’s Intelligence Scale for Indian Children and Trail Making Test

Test	*n*	Mean	SD	df	*“t”*	Significance *“P”*
Neurodevelopment						
COA	17	8.5	3.34	30	3.9	*P*<0.001
CON	15	4.1	2.17			
MISIC Scales						
Verbal						
COA	17	93.28	7.9	30	4.13	*P*<0.001
CON	15	107.1	8.7			
Performance						
COA	17	95.7	8.9	30	3.3	*P*<0.01
CON	15	108.2	9.9			
Full Scale						
COA	17	96.14	9.36	30	2.5	*P*<0.05
CON	15	105.1	7.75			
Trail Making Test						
Time						
COA	17	378.93	82.6	30	1.6	Not Significant
CON	15	311.37	127.6			
Errors						
COA	17	6.3	3.7	30	3.6	*P*<0.001
CON	15	1.8	2.18			

COA - Children of alcohol-dependent fathers; CON - Children of normals

Neuropsychological functioning in terms of general intelligence and perceptuo motor skills was assessed using the MISIC and the TMT. The results of these two tests are presented in Table 4.

As can be seen from the table, there is a statistically significant difference between the two groups on the Verbal, Performance as well as Full scale IQ scores on the MISIC.

In the TMT, there was a statistically significant difference between the two groups in the terms of the errors made – children of alcohol-dependent parents made more errors although they took a similar amount of time to do the task. The results are presented in Table 4.

Table 5 presents the data obtained from the FES. Families of alcohol-dependent parents had lower degrees of independence for its members, fewer cultural/intellectual activities and greater degree of perceived control.

**Table 5:** Scores obtained by the two groups on the various subscales of the Family Environment Scale

Scales	*n*	Mean	SD	df	*“t”*	Significance
Relationship dimension						
Cohesion						
COA	17	5	1.73	30	0.14	NS
CON	15	4.9	1.3			
Expressiveness						
COA	17	4.6	2.1	30	0.51	NS
CON	15	4.8	1.1			
Conflict						
COA	17	4.6	1.76	30	0.99	NS
CON	15	5.2	1.42			
Personal development/personal growth dimension						
Independence						
COA	17	3.9	1.85	30	2.79	*P*<0.01
CON	15	5.6	0.92			
Achievement orientation						
COA	17	4.9	2.2	30	0.21	NS
CON	15	5.1	1.5			
Intellectual/cultural orientation						
COA	17	3.2	1.2	30	4.1	*P*<0.001
CON	15	5.5	1.43			
Moral/religious						
COA	17	3.76	2.6	30	0.24	NS
CON	15	4.00	1.8			
Active/recreational						
COA	17	5.53	1.7	30	0.52	NS
CON	15	5.9	1.75			
System maintenance						
Dimension						
Organization						
COA	17	6.07	1.8	30	0.6	NS
CON	15	6.54	2.02			
Control						
COA	17	4.46	1.6	30	2.1	*P*<0.05
CON	15	3.18	1.4			

COA - Children of alcohol-dependent fathers; CON - Children of normals

## DISCUSSION

The results can be discussed by answering three broad questions:

1. Behaviorally, are children of alcohol-dependent fathers different from children of non–alcohol-dependent fathers?
2. Do children of alcohol-dependent fathers have difficulties with cognitive tasks?
3. Are family environments in families with an alcohol-dependent father different from normal families?

There were no age or sex differences between the two groups.

1. In this study, we used the CBCL as a tool for assessment of behavioral problems. We found that there were no significant differences between the two groups on the Internalizing subscale. On the Externalizing subscale, there was a statistically significant difference between the two groups. Other authors have also found, with larger samples, that children of alcohol-dependent parents, especially daughters of alcohol-dependent parents, scored higher than controls on the CBCL – both on the Internalizing and Externalizing Scales.[7,9,10] In Indian literature too, studies have shown that there is higher externalizing behavior in children who are at high risk for alcohol dependence compared to those at low risk.[2] Behavioral and emotional difficulties are commonly seen in children of alcohol-dependent parents and substantive findings exist in literature. Even with a small sample, our data Corroborates this. It was found that children had particular difficulties with handling frustration, were irritable and overall described by parents as children needing extra supervision. This is an issue that can be due to both intrinsic as well as extrinsic reasons. Genetic studies have shown that children of alcohol-dependent fathers tend to be temperamentally more difficult from infancy and disciplining patterns also tend to be inconsistent in such families. This causative link needs further exploration.
2. To assess cognitive functioning, we used several unrelated and probably overlapping measures.
  The ‘Neurodevelopmental Scale’ has been used previously to assess gross cognitive dysfunction in children at risk (having various neonatal problems particularly low birth weight and preterm babies). In this study, we used this measure as a gross indicator of cognitive functioning. This scale has shown significant differences between the children of alcohol-dependent parents and normals, with the children of alcohol-dependent parents scoring higher (higher scores indicate greater difficulty with neurodevelopmental tasks). This is an interesting finding as most studies in this area have looked at specific skills.[2,3,13,14] Although this is a gross indicator, the fact that a significant difference was found even on this scale reflects the nature and extent of the deficits.
  The TMT, a test of controlled attention, is a sensitive measure of frontal dysfunction. In this test, two aspects were considered – the time taken and the errors made. As can be seen from the data, there were significant differences between the two groups, that is, children of alcohol-dependent parents took as much time to do the task as the control group but tended to make more errors and this difference is statistically significant. This decision of choosing speed over accuracy is a function of the frontal lobe and frontal dysfunction is well known in children of alcohol-dependent parents.[3,11–13] Our study proves this as well. In addition, as seen on the CBCL, impulsivity was also found to be a significant factor differentiating children of alcohol-dependent parents from normal children. This impulsivity may also be reflected in the choice of doing a task quickly rather than accurately.
  On the MISIC, there is a statistically significant difference on the Verbal, Performance and Full Scale IQ with children of alcohol-dependent parents obtaining lower scores than normal controls, indicating a global dysfunction. Assessment of cognitive dysfunction using the Wechsler scales has shown significant differences in previous studies as well.[12] The reason for this has been discussed in some studies – some explanations are related to the general level of stimulation at home, some to the educational level of parents, some to more basic neuropsychological dysfunction. This study, however, is based on a small sample and cannot make causative links but this is an area that needs further research.
3. There have been a number of studies focusing on various aspects of family environment.[4,7,9] We found, in our study, that statistically significant differences were present on several aspects of family functioning. In the families where the father was alcohol dependent, there appeared to be decreased independence for its members, fewer opportunities for cultural and intellectual activities and there was a greater degree of perceived control when compared to normal families. The lack of independence and the greater degree of perceived control are obviously linked. Family members may feel dictated to. This has also been a finding by other authors[4,7] who reported that fathers would be aggressive, impose their will on others and did not tolerate non compliance especially in the family context. The other dimension where there is a significant difference between the families of alcohol-dependent parents and normal controls is the dimension of cultural and intellectual activities. This has not been discussed in many studies. It appears, in our study, that family members felt that fathers who were alcohol dependent were not available for family rituals and social interactions. Drinking related behaviors may take precedence over other activities. This is definitely an area where children and their mothers have reported, in our study, to be of some concern to them. Therefore, this seems to be an area that will need clinical attention to ensure that families’ coping styles are adaptive. This is discussed in some detail in another study from India.[4]

Therefore, this adds to an extensive body of literature showing that there is increased psychopathology in the form of increased externalizing behavior, difficulties in neurodevelopment and frontal lobe functions and some differences in the family environment in children and families of alcohol-dependent fathers when compared to normal controls.

## CONCLUSIONS

There is enough evidence in literature to indicate that children in families where there is an alcohol-dependent father are at increased risk for behavioral and emotional problems, cognitive deficits and dysfunctional family environment. Our study endorses these findings in the Indian context. In addition, this is a group at risk and preventive strategies in the form of early assessment and intervention for possible problem areas would definitely be helpful.
